# A Luminescent 1D Silver Polymer Containing [2.2]Paracyclophane Ligands

**DOI:** 10.3389/fchem.2021.728845

**Published:** 2021-08-02

**Authors:** Campbell F. R. Mackenzie, Lucie Delforce, D. Rota Martir, David B. Cordes, Alexandra M. Z. Slawin, Eli Zysman-Colman

**Affiliations:** Organic Semiconductor Centre, EaStCHEM School of Chemistry, University of St Andrews, Fife, United Kingdom

**Keywords:** coordination polymer, cyclophane, supramolecular chemistry, silver(I), paracyclophane

## Abstract

[2.2]Paracyclophane scaffolds have seen limited use as building blocks in supramolecular chemistry. Here, we report the synthesis and characterization of a 1D coordination polymer consisting of silver(I) ions bound to a [2.2]paracyclophane scaffold functionalized with two 4-pyridyl units. The structure of the polymer has been determined from single crystal X-ray diffraction analysis and reveals two different silver coordination motifs that alternate along the 1D coordination polymer. The coordination polymer exhibits strong blue and sky-blue fluorescence in solution and in the crystalline solid state, respectively.

## Introduction

Coordination polymers are polymeric arrays of monomeric units that are held together through metal coordination (Yang et al., 2015). These materials continue to attract attention due to their use in a wide range of applications from medicine (Medici et al., 2016), to catalysis (Zeng et al., 2016), sensors (Yi et al., 2016) and luminescent materials (Yersin et al., 2017) (To et al., 2020). Among the metals used to synthesize coordination polymers, silver is of particular interest due to the range of geometries and coordination modes available (Young and Hanton, 2008). Silver has been demonstrated to form coordination polymers with a range of morphologies, 1D, 2D and 3D coordination networks have been demonstrated for a range of ligands (Carlucci et al., 1995) (Chen et al., 2006) (Roy et al., 2016).

Luminescent coordination polymers containing silver(I) were first documented in 1999 (Tong et al., 1999) with the report of a self-assembled 3D coordination polymer of silver(I) with bis-phenol Schiff base ligands that showed bright blue emission in both the solid state and in solution. Since then, many luminescent silver coordination polymers have been reported. Macrocycles and 2D structures made of Ag(I) and pyrimidine-based thioether ligands, reported by Han and coworkers (Han et al., 2005), also show bright luminescence. Liu and coworkers (Liu et al., 2005) reported 2D networks with metallocyclophane motifs with high electric conductivity due to columnar aromatic stacking motifs formed through both intra- and intermolecular π−π interactions. They also report a 2D zigzag sheet structure, in which silver triflate forms tubelike double chains with 4,4′-bis (2,5-dimethylstyryl) biphenyl molecules acting as links between the chains to form the sheets. Both of those compounds exhibit luminescence in the solid state and show excitation and emission maxima are shifted to longer wavelength as compared to those of the corresponding metal-free ligands. The same observation was made by Huo and coworkers (Huo et al., 2016) who reported a series of luminescent Ag(I) coordination polymers with different coordination modes tuned via different multidentate bis(1,2,3-triazole) ligands and anions. This anion-responsive behavior was also detected by Fan and coworkers (Fan et al., 2014) in 3D porous luminescent triazol-type Ag(I) framework with green luminescence emission. Co-crystallization of Ag(I) complexes with bipyridine and benzimidazole was reported by Cai and coworkers (Cai et al., 2011) to yield 1D infinite coordination polymer chains with both intraligand emission and ligand-to-metal charge transfer contribution. We reported the first example of a phosphorescent Ag(I) coordination polymer that incorporated iridium (III) metalloligands (Rota Martir et al., 2018).

The two benzene rings of the [2.2]paracyclophane (pCp) are disposed cofacially (Bahrin et al., 2017, 2), which give rise to through-space (π–π *trans*-annular) and through-bond [σ(bridge)–π(annular)] electronic interactions, affecting the chemical, optical, and electronic properties of the molecule (Kahnt et al., 2007; Elacqua and MacGillivray, 2010). It has been shown that positioning organic functional groups on the pCp core ring shifts its luminescence properties (Braun et al., 2017; Anhäuser et al., 2019; Rota Martir et al., 2019). Exploiting the electronic coupling present between the two benzene decks of the pCP, the first examples of pCp compounds emitting via a thermally activated delayed fluorescence mechanism were recently reported by Spuling, Sharma and co-workers (Spuling et al., 2018). There are few examples of the pCp scaffold used in metallosupramolecular self-assembly (Meyer-Eppler et al., 2014; Gon et al., 2017; Anhäuser et al., 2019; Rota Martir et al., 2019). A [Pd_3_(pCpd4py)_6_](BF_4_)_6_ 3D-photoactive cage assembly incorporating a pyridyl-substituted pCp scaffold and Pd has been reported (Rota Martir et al., 2019), this macrocycle is emissive in both the solid state and solution, with emission red-shifted relative to the ligand.

Herein we report the synthesis and photoluminescence properties of a remarkable 1D coordination polymer containing silver(I) ions in two different coordination environments. The coordination polymer Ag-pCp was obtained from the reaction of Ag(I) ions and a [2.2]paracyclophane functionalized at the 7 and 15-positions with 4-pyridyl moieties (pCpd4py).

## Methods

### Synthesis

The ligand pCpd4py was prepared according to a previously published method (Rota Martir et al., 2019). All other chemicals and solvents were obtained from commercial suppliers and used as received, solvents used for photophysical measurements were of spectroscopic grade.

*Synthesis of Ag-pCp polymer.* In a small vial, pCpd4py (4.0 mg, 0.01 mmol, 1 equiv.) was dissolved in 1 ml of dichloromethane. To this vial a solution of AgPF_6_ (10 mg, 0.04 mmol, 3.6 equiv.) in 1 ml of acetonitrile was carefully layered on top. The vial was sealed and left at room temperature, crystals began forming after 3 days and the reaction was complete after 10 days. The colorless crystals were collected to give the product (5.0 mg, 52% yield). The product was identified by single crystal X-ray diffraction, with the formula determined to be [Ag_3_(pCpd4py)_4_(NCCH_3_)_6_(CH_2_Cl_2_)_2_] (CCDC: 2089070).

### Photophysics

Absorption spectra were recorded at room temperature on a Shimdazu-1800 spectrophotometer in 1 cm quartz cuvettes. For emission studies, degassed solutions were prepared via three freeze-pump-thaw cycles and spectra were taken using a home-made Schlenk quartz cuvette. Crystalline samples were finely ground and mounted in a quartz sample holder for solid-state measurements. Steady-state emission, excitation spectra and time-resolved emission spectra were recorded at 298 K using an Edinburgh Instruments F980 fluorimeter. Samples were excited at 378 nm using a pulsed diode laser for time-resolved measurements.

## Results and Discussion

Coordination of silver ions to the 4-pyridyl groups of pCpd4py proceeded readily, with the coordination polymer crystallizing from solution cleanly in moderate yield.

**FIGURE 1:**
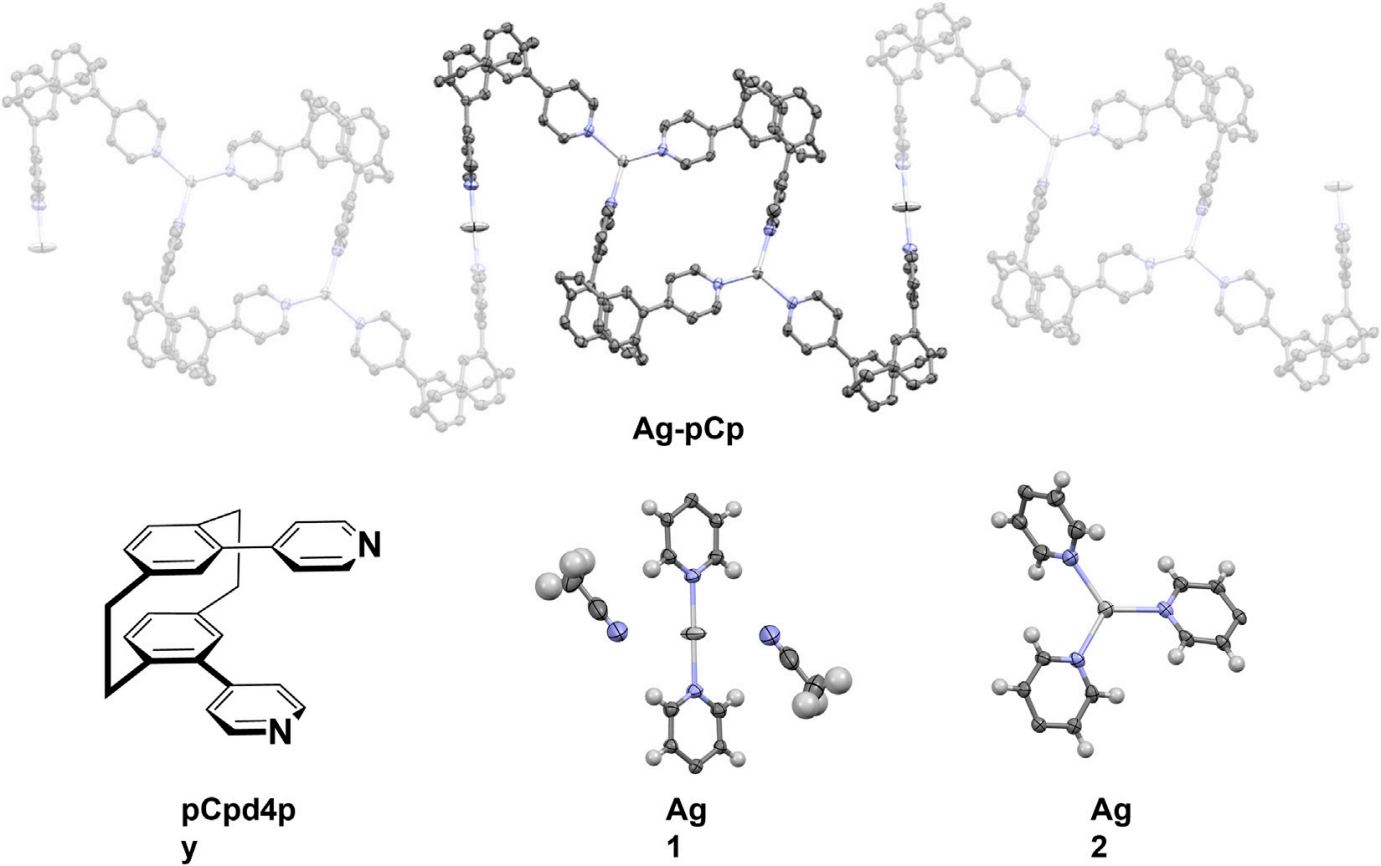
(Top) Representation of the X-ray crystal structure of Ag-pCp, hydrogen atoms, solvent molecules and anions are omitted for clarity. (Bottom, L-R) Diagram of ligand pCpd4py, two different coordination environments for the silver atoms (thermal ellipsoid plot, 50% probability ellipsoids).

The X-ray crystal structure of the coordination polymer is shown in Figure 1. The asymmetric unit of the structure comprises one and a half silver(I) ions, two pCp4py ligands, one and a half PF_6_^–^ anions, one molecule of dichloromethane solvent and three molecules of acetonitrile solvent. This remarkable structure consists of a 1D coordination polymer in which there are two different coordination environments for the silver(I) ions. One silver ion (Ag1) is in a linear, 2-coordinate environment, while the second silver ion (Ag2) is in a trigonal planar 3-coordinate environment.

The linear coordination environment around Ag1 has two strongly bound nitrogen atoms from the pCpd4py ligand. Ag1 is located at an inversion center, this results in a perfect 180° N-Ag1-N bond angle and identical Ag1-N distances of 2.141(4) Å. A linear N-Ag-N bond has been observed in some Ag-coordination polymers, for example in *rac-*IrAg (Rota Martir et al., 2018); however, in many cases the linear coordination is distorted by the presence of anions and solvent molecules (Chen et al., 2006). In the extended coordination sphere, there are two weakly bound acetonitrile molecules, with longer Ag to N distances of 2.833(10) Å. The weakly coordinated acetonitrile molecules are nearly perpendicular to the Ag-N_py_ bonds, with a N_py_-Ag1-N_MeCN_ angle of 87.6(3) The Ag1-N distance in Ag-pCp is similar to the 2.141 and 2.156 Å observed in *rac* **-**IrAg (Rota Martir et al., 2018) and also similar to the Ag-N distances of between 2.123 and 2.133 Å seen in a series of linear two-coordinate [Ag (pyridine)_2_]^+^ structures. (Chen et al., 2007).

The coordination environment around Ag2 is a nearly perfect trigonal planar environment, with Ag2-N bond lengths of 2.224(4), 2.246(5) and 2.268(4) Å and N-Ag2-N angles between 112.85(16) and 124.31(17), with the Ag(I) ion displaced 0.099 Å out of the mean-plane of the nitrogen atoms. This is similar to the coordination environment seen in a pair of [Ag(isonicotinamide)_3_]^+^ crystals where Ag-N bond lengths ranged between 2.213 and 2.321 Å, N-Ag-N bond angles were *ca*. 105, 120 and 135°, and the Ag(I) ion was displaced 0.007 or 0.017 Å out of the mean-plane of the nitrogen atoms (B. Aakeröy et al., 1998). A three-coordinate geometry for silver is the third most common seen, behind four- and two-coordinate (Young and Hanton, 2008), in many of these examples the coordination environment is distorted from the ideal trigonal planar, trigonal pyramidal or T-shaped geometries by the use of bidentate ligands or the presence of weak interactions with solvent molecules or anions (Durini et al., 2017). The angled orientation of the coordinating pyridyl groups makes each Ag2 center a chiral molecular propeller, although from the symmetry of the space group, centers of both handedness are present and individual polymer chains are achiral.

The phenyl rings of the pCp4py ligands show a distortion from planarity similar to that seen in related pCp compounds (angle across the *para*-substitution axis 15.6–16.2°). (Rota Martir et al., 2019). Despite this, the dihedral angle between mean planes of the phenyl groups still shows them to be parallel (1.8° for both ligands). Each ligand shows two rather different dihedral angles between the phenyl and its pyridyl substituent, although these match reasonably well between the two ligands (31.6 and 49.2° for one, 36.9 and 51.7° for the other). In turn, this leads to similar angles between pyridyl rings for each of the two ligands (75.4 and 83.2°). This matches the conformation seen in other pyridyl-cyclophanes. (Rota Martir et al., 2019).

**FIGURE 2:**
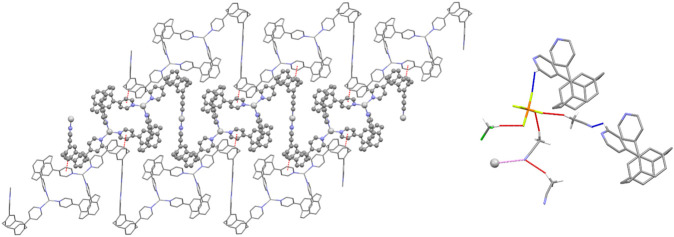
Representations of the intermolecular interactions in the solid-state structure of Ag-pCp. (Left) View of a two-dimensional sheet in the (1 1 1) plane formed by C-H·π interactions (shown red) linking adjacent chains. The central chain is highlighted for clarity. (Right) View of the weakly interacting T-shaped motif (interactions shown red) formed by solvent and anion. Links from the motif to polymer chains are shown for both C-H···A (shown blue) and Ag···N (shown pink) interactions.

Each polymer chain is built from alternating [Ag_2_(pCpd4py)_2_] 32-membered metallamacrocycles (which include just the trigonal planar Ag2) and Z-shaped linkers, comprising two ligands bound linearly to Ag1. This results in a flat, tape-like coordination polymer (Figure 1), with chains propagating along the [0 1 −1] diagonal axis, and a polymer repeat-distance of 21.42 Å. Adjacent polymer chains are offset such that the edge of one tape overlies that of the next, with the pCp of the Z-shape above the trigonal planar Ag2 center. Despite this positioning, no π···π interactions are found in the structure, the shortest distance between centroids being 4.33 Å. However, this positioning of adjacent chains does allow for the formation of CH···π interactions chains, involving a methylene hydrogen of one pCpd4py and the π-system of a pyridine ring of the other, at a C-H···centroid distance of 2.70 Å [C···centroid separation of 3.528(6) Å]. These interactions occur in pairs across an inversion center, and link adjacent chains into two-dimensional sheets in the (1 1 1) plane (Figure 2). No direct intermolecular interactions occur between adjacent sheets. There are no close contacts between the silver(I) atoms, in contrast to many low-coordinate silver complexes. (Chen et al., 2007).

Both anions and solvent molecules form weak C-H···A interactions, although only the PF_6_^–^ anions and one of the molecules of acetonitrile interact directly with the polymer chains, other interactions being between anion and solvent or solvent and solvent. None of the anions or solvent molecules bridge directly between chains. The C-H···N distances occur at 2.47 and 2.61 Å [C···N separations of 3.371(15) and 3.24(2) Å] and the C-H···F distances range from 2.40 to 2.45 Å [corresponding C···F separations of 3.150(9) to 3.400(14) Å]. One PF_6_^–^ interacts solely with one polymer chain, while the other interacts with a chain and multiple solvent molecules. These weak interactions give rise to discrete T-shaped motifs comprising one dichloromethane, one PF_6_^–^ and three acetonitriles (Figure 2). Two polymer chains in the same sheet are weakly linked via the PF_6_^–^ and one acetonitrile, while this sheet can in turn be linked to a chain in another sheet via the weak Ag···NCMe interactions.

**FIGURE 3:**
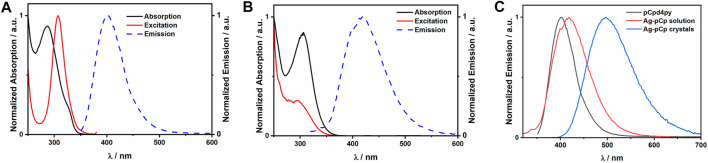
Plots of the normalized absorption, excitation and emission spectra for **(A)** pCpd4py in acetonitrile, **(B)** Ag-pCp in dichloromethane. **(C)** Comparison of the emission spectra for the ligand and Ag-pCp in solution and crystalline states.

Ag-pCp is luminescent, exhibiting sky-blue emission (λ_PL_ = 497 nm) in the crystalline solid state and deep-blue emission in DCM solution (λ_PL_ = 419 nm), Figure 3. Coordination polymers are liable to exist as shorter oligomers in solution, although in a non-coordinating solvent like DCM, Ag-pCp is expected to retain its structural motifs in the oligomeric fragments. The solution-state emission is only slightly red-shifted from the emission of the ligand (λ_PL_ = 403 nm), suggesting that the emission is fluorescence from a ligand centered (^1^LC) singlet excited state, with the red-shift due to the stabilization of the LUMO and the singlet excited state upon coordination of the Lewis acidic silver(I) ions to the pyridyl group of the ligand. This same red-shift in emission upon coordination of a metal ion to pCp-d4py was seen for [Pd_3_(pCpd4py)_6_](BF_4_)_6_. (Rota Martir et al., 2019). There is a similar red-shift observed in the absorption spectrum of a DCM solution of Ag-pCp (λa_bs_ = 306 nm) in comparison to the free ligand (λ_abs_ = 287 nm). The emission lifetime of pCpd4py and the DCM solution of Ag-pCp are very similar (pCpd4py: τ_PL_ = 5.42 ns, Ag-pCp: τP_L_ = 5.27 ns), while crystalline Ag-pCp has a longer emission lifetime of 19 ns.

**FIGURE 4:**
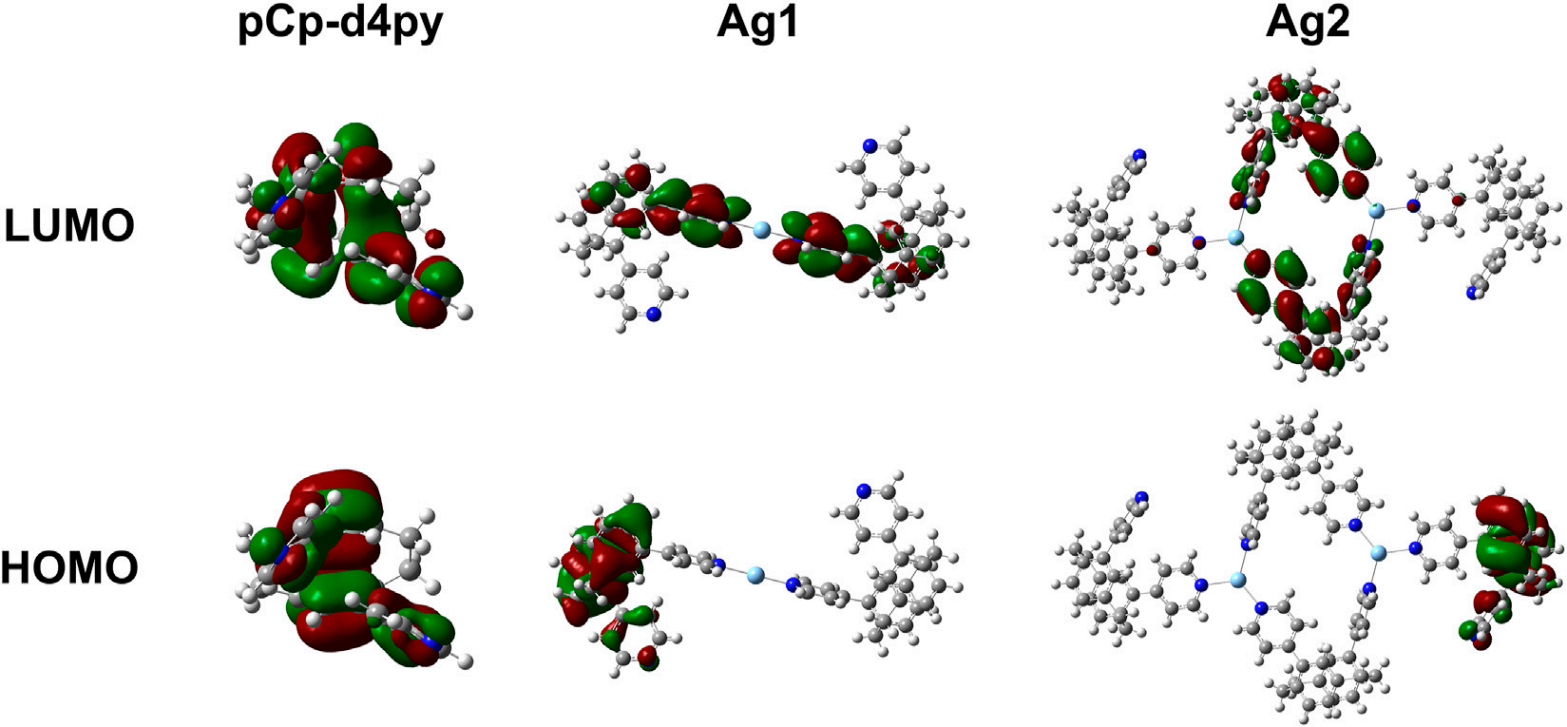
Plots of the molecular orbitals (top–LUMO, bottom–HOMO) for pCpd4py and the two different Ag-containing model units from Ag-pCp.

The electronic properties of both the ligand and subunits of the polymer were investigated by DFT and TD-DFT calculations to provide further insight into the nature of the emission properties and the corresponding energy levels of the excited states of the compounds. Details of the calculations performed are provided in the Supplementary Material, while plots of the Kohn-Sham orbitals are shown in Figure 4. The optimized structure of the ligand showed a large gap of 4.50 eV between the HOMO and LUMO, which is also reflected in the high excited state energy of 3.77 eV for S_1_. There is no spatial separation between the HOMO and LUMO, leading to a large singlet-triplet energy gap, ΔE_ST_, of 0.89 eV.

We next investigated Ag-pCp. The polymer was broken down into two different chromophoric units that were studied separately. The first unit contained a single two-coordinate silver center with the two coordinating pCpd4py ligands (matching the environment of Ag1). The second unit contained two three-coordinate silver centers, with two pCpd4py ligands bridging the Ag ions and forming a metallamacrocycle, and two additional ligands completing the coordination sphere of each Ag center (matching Ag2). The optimized geometry for each silver environment is similar to the crystallographically determined structures. The optimized Ag1 structure has a linear, two-coordinate environment around the silver ion, with the two pyridine groups being co-planar. The Ag2 structure retains the trigonal planar coordination environment observed experimentally, although the environment is slightly distorted, with py-Ag-py bond angles ranging from 94 to 140° and the Ag-N_py_ distances ranging from 2.238 to 2.395 Å with the silver ions displaced up to 0.229Å from the mean-plane of the nitrogen atoms.

The coordination of the silver ions to the ligand in each of these units resulted in a strong stabilization of more than 50 meV on the LUMO of the compounds, with little change observed in the HOMO energy. The HOMO remaining localized on a single ligand, while the LUMO is distributed across multiple ligands. In all cases there is no metal contribution to the orbitals (Figure 3).

The smaller Ag1-type unit shows a significant (50 meV) red-shift in the excited state energy of S_1_, while the larger Ag2-type unit shows a more moderate 20 meV red-shift of the emissive excited state. The calculated red-shift for the Ag2-containing fragment is close to the 11 meV red-shift observed between the solution state emission of the ligand in MeCN and that of the polymer in DCM. This suggests that the Ag2-type group is a good computational model for the whole polymer in solution.

## Conclusion

We have prepared a new luminescent 1D silver(I) coordination polymer Ag-pCp. The polymer has a very interesting structure, containing both linear 2-coordinate and trigonal planar 3-coordinate silver atoms in the polymer chain. Ag-pCp is blue fluorescent in both the crystalline state and in DCM solution.

## Data Availability

The datasets presented in this study can be found in online repositories. The names of the repository/repositories and accession number(s) can be found below: https://doi.org/10.17630/9e0d4b83-171a-4805-aef8-a9fbac035c1f.
