# Supplementary material for: A Luminescent 1D Silver Polymer Containing [2.2]Paracyclophane Ligands
Source: Front Chem. 2021 Aug 2;9:728845. doi: 10.3389/fchem.2021.728845 (PMC8371969; doi:10.3389/fchem.2021.728845)
Supplement: Supplementary file 1 [file datasheet1.pdf]

frontiers

# A Luminescent 1D Silver Polymer Containing [2.2]Paracyclophane Ligands

## *Supplementary Material*

## 1 Crystallographic Data

X-ray diffraction data for **Ag-pCp** were collected at 173 K using a Rigaku FR-X Ultrahigh Brilliance Microfocus RA generator/confocal optics with XtaLAB P200 diffractometer [Mo Kα radiation (λ = 0.71075 Å)]. Intensity data were collected using ω steps accumulating area detector images spanning at least a hemisphere of reciprocal space. Data were collected and processed (including correction for Lorentz, polarization and absorption) using CrystalClear (CrystalClear-SM Expert v2.1, 2015). The structure was solved by Patterson methods (PATTY) (Beurskens et al., 1999) and refined by full-matrix least-squares against $F^2$ (SHELXL-2018/3) (Sheldrick, 2015). Non-hydrogen atoms were refined anisotropically, and hydrogen atoms were refined using a riding model. All calculations were performed using the CrystalStructure (CrystalStructure v4.3.0, 2018) interface. Deposition number 2089070 contains the supplementary crystallographic data for this paper. These data are provided free of charge by the joint Cambridge Crystallographic Data Centre and Fachinformationszentrum Karlsruhe Access Structures service www.ccdc.cam.ac.uk/structures.

Crystal data. $C_{118}H_{110}Ag_3Cl_4F_{18}N_{14}P_3$, $M$ = 2624.57, triclinic, $a$ = 12.2855(11), $b$ = 14.2571(13), $c$ = 17.4718(18) Å, $\alpha$ = 84.250(9), $\beta$ = 70.119(6), $\gamma$ = 76.639(8) °, $U$ = 2799.2(5) Å$^3$, $T$ = 173 K, space group $P\bar{1}$ (no. 2), $Z$ = 2, 33170 reflections measured, 10036 unique ($R_{int}$ = 0.0390), which were used in all calculations. The final $R_1$ [$I > 2\sigma(I)$] was 0.0683 and $wR_2$ (all data) was 0.1971.

## 2 DFT Calculations

DFT calculations were performed using the Gaussian16 (Rev. C.01) suite of software (Frisch et al., 2016). All calculations were performed using the B3LYP functional (Lee et al., 1) and the 6-31G(d,p) basis set (McLean and Chandler, 1980) for non-metal atoms and the SBKJC VDZ ECP basis set (Binkley et al., 1980) for Ag atoms, using the conductor-like polarizable continuum model (CPCM) for dichloromethane solution (Barone and Cossi, 1998; Cossi and Barone, 2001; Cossi et al., 2003).

The crystallographic structure of **Ag-pCp** was used at the starting point for ground state geometry optimizations, the polymer was broken down into **pCpd4py**, **Ag1** or **Ag2** units in Gaussview v6.0 (Dennington et al., 2019). Vibrational frequency calculations were performed on all optimized structures to ensure that the optimized geometries represented the local minima. Excited state energies were calculated using the TD-DFT formalism (Casida et al., 1998; Stratmann et al., 1998). Calculated structures and Kohn-Sham orbitals were visualized with Gaussview v6.0 (Dennington et al., 2019).

## 3 References

Barone, V., and Cossi, M. (1998). Quantum Calculation of Molecular Energies and Energy Gradients in Solution by a Conductor Solvent Model. *J. Phys. Chem. A* 102, 1995–2001. doi:10.1021/jp9716997.

Beurskens, P. T., Beurskens, G., de Gelder, R., Garcia-Granda, S., Gould, R. O., Israel, R., et al. (1999). *DIRDIF-99*. The Netherlands: Crystallography Laboratory, University of Nijmegen.

Binkley, J. S., Pople, J. A., and Hehre, W. J. (1980). Self-consistent molecular orbital methods. 21. Small split-valence basis sets for first-row elements. *J. Am. Chem. Soc.* 102, 939–947. doi:10.1021/ja00523a008.

Casida, M. E., Jamorski, C., Casida, K. C., and Salahub, D. R. (1998). Molecular excitation energies to high-lying bound states from time-dependent density-functional response theory: Characterization and correction of the time-dependent local density approximation ionization threshold. *J. Chem. Phys.* 108, 4439–4449. doi:10.1063/1.475855.

Cossi, M., and Barone, V. (2001). Time-dependent density functional theory for molecules in liquid solutions. *J. Chem. Phys.* 115, 4708–4717. doi:10.1063/1.1394921.

Cossi, M., Rega, N., Scalmani, G., and Barone, V. (2003). Energies, structures, and electronic properties of molecules in solution with the C-PCM solvation model. *J. Comput. Chem.* 24, 669–681. doi:10.1002/jcc.10189.

CrystalClear-SM Expert v2.1 (2015). and Rigaku Corporation,Tokyo, Japan: Rigaku Americas, The Woodlands, Texas, USA.

CrystalStructure v4.3.0 (2018). and Rigaku Corporation,Tokyo, Japan: Rigaku Americas, The Woodlands, Texas, USA.

Dennington, R., Keith, T., and Millam, J. (2019). *Gaussview, Version 6*. Shawnee Mission KS: Semichem Inc.

Frisch, M. J., Trucks, G. W., Schlegel, H. B., Scuseria, G. E., Robb, M. A., Cheeseman, J. R., et al. (2016). *Gaussian 16 Rev. C.01*. Wallingford, CT.

Lee, C., Yang, W., and Parr, R. G. (1). Development of the Colle-Salvetti correlation-energy formula into a functional of the electron density. *Phys. Rev. B* 37, 785–789. doi:10.1103/PhysRevB.37.785.

McLean, A. D., and Chandler, G. S. (1980). Contracted Gaussian basis sets for molecular calculations. I. Second row atoms, Z=11–18. *J. Chem. Phys.* 72, 5639–5648. doi:10.1063/1.438980.

Sheldrick, G. M. (2015). Crystal structure refinement with SHELXL. *Acta Crystallogr. C* 71, 3–8. doi:10.1107/S2053229614024218.

Stratmann, R. E., Scuseria, G. E., and Frisch, M. J. (1998). An efficient implementation of time-dependent density-functional theory for the calculation of excitation energies of large molecules. *J. Chem. Phys.* 109, 8218–8224. doi:10.1063/1.477483.
